# Effects of acupuncture treatment on depression insomnia: a study protocol of a multicenter randomized controlled trial

**DOI:** 10.1186/1745-6215-14-2

**Published:** 2013-01-03

**Authors:** Yuan-Fang Chen, Jian-Hua Liu, Neng-Gui Xu, Zhao-Hui Liang, Zhen-Hua Xu, Shu-Jun Xu, Wen-Bin Fu

**Affiliations:** 1 Guangzhou University of Traditional Chinese Medicine (TCM), Guangzhou, China; 2 General Acupuncture Department of Guangdong Provincial Hospital, Guangzhou, China

## Abstract

**Background:**

More than 70% of patients with depression who see their doctors experience insomnia. Insomnia treatment is a very important link for depression treatment. Furthermore, antidepression treatment is also important for depression insomnia. In acupuncture, LU-7 (Lie Que) and KID-6 (Zhao Hai), which are two of the eight confluence points in meridian theory, are used as main points. An embedded needle technique is used, alternately, at two groups of points to consolidate the treatment effect. These two groups of points are BL-15 (Xin Shu) with BL-23 (Shen Shu) and BL-19 (Dan Shu) with N-HN-54 (An Mian). The effectiveness of these optimized acupuncture formulas is well proven in the practice by our senior acupuncturists in Guangdong Provincial Hospital of TCM. This study has been designed to examine whether this set of optimized clinical formulas is able to increase the clinical efficacy of depression insomnia treatment.

**Methods/design:**

In this randomized controlled multicenter trial, all the eligible participants are diagnosed with depression insomnia. All participants are randomly assigned to one of two groups in a ratio of 1:1 and receive either conventional acupuncture treatment or optimized acupuncture treatment. Patients are evaluated using the Pittsburgh Sleep Quality Index(PSQI)and the Hamilton rating scale(HAMD) for depression. The use of antidepression and hypnotics drugs is also considered. Results are obtained at the start of treatment, 1 and 2 months after treatment has begun, and at the end of treatment. The entire duration of the study will be approximately 36 months.

**Discussion:**

A high quality of trial methodologies is utilized in the study, and the results may provide better evidence for the effectiveness of acupuncture as a treatment for depression insomnia. The optimized acupuncture formula has potential benefits in increasing the efficacy of treating depression insomnia.

**Trial registration:**

The trial was registered in Chinese Clinical Trial Register (ChiCR-TRC-00000481) on 12 August 2009.

## Background

More than 20% of Chinese people with depression and depressive neurosis experience from depressive symptoms [1]. Depression is a mental disorder with various characteristics, including negative emotions, anxiety, agitation, low self-esteem, dizziness, and suicidal tendencies [2]. Excessive levels of disturbed sleep among patients form one of the core symptoms known to be related with depression [3,4]. The prevalence of patients with depression and insomnia varies from 70.0% to 84.7%. The Insomnia is likely to increase the risk of deterioration and recurrence of depression, heart disease, social functioning, and the risk of suicide. Moreover, insomnia reduces the quality of life of patients with depression [5,6]. Meanwhile, insomnia is one of the most-diagnosed symptoms of depression and can be a risk factor for intractable depression and a predictor of depression disorder recovery [7]. Therefore, for those patients with depression insomnia, insomnia cannot simply be addressed through the use pharmacological treatment or other therapies for insomnia or depression. Treatments for both are very important.

Acupuncture, as a typical traditional Chinese medicine (TCM), has been applied for thousands of years [8]. The rapid development of acupuncture both within and outside China over the last few decades has itself led to great innovations in practice. Many studies, including clinical reports and systematic reviews, have investigated the benefits and success of acupuncture in relieving symptoms for various acute and chronic diseases, while a large history of clinical practice demonstrates that acupuncture appears to have an irreplaceable effect in treating depression and its symptoms, when compared with modern medicine and its antidepressive treatment. However, methodological flaws in design undermine the validity of these studies [9-11]. For example, two systematic reviews by Smith in the Cochrane Collaboration show that the clinical evidence for acupuncture treatment of depression is relatively limited before 2003 and that the quality of the clinical trials has improved between 2003 and 2008. The curative effects of acupuncture for depression are impossible to confirm [12,13].

In view of these data,this randomized, controlled, multicenter study of depression insomnia patients aims to determine the effectiveness of traditional acupuncture techniques. The study will compare a conventional acupuncture treatment formula with an optimized acupuncture treatment formula. The conventional acupuncture treatment formula is based on a point selection established on the basis of the particular TCM pattern of depression. Depression in Chinese medicine is mainly due to liver Qi stagnation, giving rise to symptoms appearing to be mental disturbance. LI-4 (He Gu), LIV-3(Tai Chong), EX-HN-3 (Yin Tang), and GV-20 (Bai Hui) are the points of the conventional acupuncture treatment formula. In optimized acupuncture formulas, the two empirical points LU-7 (Lie Que) and KID-6 (Zhao Hai) are added to the conventional acupuncture treatment formula. The action of these two points is used to improve the sleep quality of patients. Meanwhile, an embedded needle technique has proved to be effective for chronic disease or for those diseases demanding a longer retention time. Therefore, the technique is used to extend the effective treatment period in optimized acupuncture formulas [14].

A measurement system for evaluating the progression of illness, such as sleep quality and stress levels, will be used to compare the exact effect of different acupuncture treatment formulas.

## Methods/design

### Trial objective

The objective of this trial is to evaluate the effectiveness of treating depression insomnia by comparing the effects of a conventional acupuncture treatment formula and an optimized acupuncture treatment formula.

### Hypothesis

Acupuncture is capable of reducing levels of depression, whether with a conventional treatment formula or an optimized treatment formula. Furthermore, optimized acupuncture is capable of reducing levels of depression insomnia, to a greater degree than conventional acupuncture formula. Meanwhile, the results of the optimized formula of acupuncture treatment for depression insomnia are supposed to increase the clinical efficacy of treating depression insomnia, improve the health-related life quality of depression insomnia patients, and moderate the consumption of drugs used in conventional treatment, eventually leading to significant social and economic benefits.

### Design

**Figure 1:**
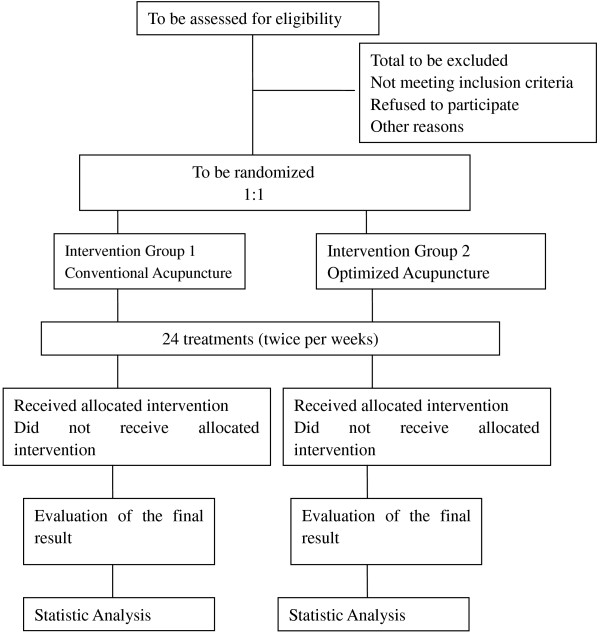
Flowchart for the study.

This is a multicenter, randomized, controlled prospective study. Participants receive optimized acupuncture (treatment group), or conventional acupuncture (control group) in a 1:1 allocation ratio (Figure 1).

#### Eligibility

Participants meeting the following criteria are included:

1. Patient meets the diagnostic criteria for depression in the Chinese Classification of Mental Disorders, 3rd edition [15].

2. Patient meets the chief diagnostic criteria for insomnia (Diagnostic and Statistical Manual of Mental Disorders, 4th edition [16].

3. The Hamilton rating scale for depression (HAMD) count should be more than 20 points but less than or equal to 35 points.

4. The total Pittsburgh Sleep Quality Index (PSQI)count should be 7 points or over.

5. Patient is conscious without aphasia and mental retardation and is able to understand the rating scales.

6. Patients must be aged at least 16 years and not more than 50 years, and may be male or female.

7. The course of disease is between 2 weeks and 2 years.

Participants meeting one or more of the following criteria are excluded:

1. The depression is caused by organic mental disorders, psychoactive drug substances, or non-addictive substances.

2. Patients are aged less than 16 years or more than 50 years.

3. Patient is unable to understand the rating scales.

4. Pregnant women, patients with unstable heart condition, liver, kidney, and hematopoietic system diseases, and those who fear acupuncture are excluded.

5. Patients who cannot cooperate with their doctors are excluded.

#### Withdrawal criteria and management

Withdrawal or dropout criteria:

1. At the participants’ own request or that of the legal representative.

2. Participants who develop a serious disease, such as heart disease or pneumonia and, in the investigator’s opinion, it is not suitable to continue.

3. The participant’s compliance is poor, in the investigators’ opinion,and it is not suitable that they should keep taking part in the research.

4. The participants have an adverse reaction related with acupuncture treatment.

Withdrawn subjects or dropouts should be managed as follows. Details of the withdrawn patients should be documented in the ‘clinical trial performance forms’ in the case report files (CRF) of the patients. Details of patients who have withdrawn as a result of an adverse event or reaction should be recorded in the CRF. If the patients who have been determined as dropout or withdrawn cases require further treatment, the investigators should provide the treatments; however, their data will be treated as missing data.Details of all withdrawn patients will reported in the final results, to guarantee maximum transparency.

#### Setting and participants

The study protocol was designed according to the Standards for Reporting Interventions in Clinical Trials Acupuncture (SRICTA) [17].

#### Sample size calculation

The sample size calculation is based on the two-sided chi-square test for difference with respect to the primary endpoint. There are few reports in the literature of clinical trialsof acupuncture treatment for depression insomnia. Based on our earlier randomized controlled pre-trial in Guangdong Provincial Hospital of TCM, we decided to adapt the reduction of the HAMD and PSQI scores as a baseline for the sample size calculation for our trial. We believe that the successful cure rate for depression insomnia is approximately 40% when adopting the conventional acupuncture formula and should be increased to 70% or more when using the optimized acupuncture formula. A sample size of 58 patients per group is necessary (as calculated using PEMS version3.1 software,with the predicted cure rates of 40% and 70%). The maximum dropout rate within the intervention is expected to be about 15%. Therefore, another 24 patients in total have to be randomized to obtain the required number of patients. The total number of patients needed to be randomized is therefore 140.

#### Recruitment of patients

The patients are referred by the doctors and acupuncturists from branch centers in Guangzhou, Huizhou, and Zhaoqing city. 70 patients are from Guangdong Provincial Hospital of TCM, 35 patients are from the second people’s hospital of Zhaoqing city, and another 35 patients are from the People’s Hospital of Huizhou city.

#### Randomizations and procedures for setting

The study procedures, risks, benefits, and data management are clarified in detail before the patients are asked to give their informed consent. To achieve comparable groups for known and unknown risk factors, a simple randomization is performed. Both groups have equal numbers of participants. The eligible participants are divided into two groups; the control group (indicated as 1 in the CRF) and the treatment group (indicated as 2 in the CRF). These groups will receive the acupuncture prescription as basic treatment but the treatment group will also be treated using the acupuncture points and techniques especially for insomnia. After inputting the estimated sample size into PEMS version 3.1, a sequence number, seed number, center number, and allocation result will be obtained. The allocation to the treatment or control group will then be performed using sealed consecutively numbered envelopes prepared by the trial team. Since this is a multicenter trial, after the patient is registered, the practitioners are informed, by telephone or other instant messenging, of the patient’s assignation to one of the two study formulas. This procedure ensures that the randomization is not influenced by the investigators taking part in this study. The practitioners participating in the study do not take part in the randomization process.

#### Blind method

All eligible participants take part in a double-blind study. The participants in both groups are blinded. The evaluation of participants and analysis of results will be performed by professionals blinded to the assignation of treatment options. Those blinded professionals are required to conduct the outcome assessments, collect medication diaries, and check any missing data. Except for the practitioners, all of those involved in the outcome assessment are blinded to participant allocation.

#### Study treatment

The patients taking part in the study will each receive 24 acupuncture sessions (twice per week), either conventional or optimized, as follows.

The treatment will be performed after sterilizing the skin on the acupuncture point areas and with patients lying face up. The temperature of the treatment room is not lower than 25°C.

1. Conventional acupuncture treatment

All the patients in intervention group 1, the control group, will receive the conventional acupuncture formula at the following points: LI-4 (He Gu), LIV-3(Tai Chong), EX-HN-3 (Yin Tang), and GV-20 (Bai Hui). The needles, size 0.35 × 25 mm,are produced by Suzhou Tianxie Acupuncture Instruments Co., Ltd. The needle are maintained in the acupuncture points for 30 minutes. During the treatment, after a De Qi sensation is produced, which is described as a numb, distended and aching sensation, patients are required to take six deep breaths and then have a rest, followed by another six deep breaths every minute until the needles are removed.

2. Optimized acupuncture treatment

The basic treatment formula is modified. On the basis of the normal acupuncture, LU-7 (Lie Que) and KID-6 (Zhao Hai) are added and needles remain in the body after patient feels the De Qi sensation. The embedded needles are then buried, for two groups of points (1) Bl-15 (Xin Shu), Bl-23 (Shen Shu), and Bl-19 (Dan Shu), N-HN-54 (An Mian); these two groups of points are used alternately. The granular type of embedded needle used has a needle tip of about 2 or 3 mm and is manufactured by Suzhou Medical Instrument Factory, China. The needle is inserted perpendicularly underneath the skin and is fixed with 3M medical proof fabric. Before the start of the next treatment, the embedded needles are removed.

To minimize treatment bias, all the acupuncturists who participate in this trial are acupuncturists of a general acupuncture department and each research center. They all gained medical licenses in China and were well trained according to the trial’s procedure under the guidance of senior acupuncturists.

### Common acupuncture adverse events or reactions and processing

#### Common acupuncture adverse events or reaction

1. Needle fainting during acupuncture treatment

For patients who are on their first visit or have a serious phobia about needles, a reassuring explanation is necessary. The patient’s complexion will be closely observed to check for any early symptoms of needle fainting, such as discomfort, dizziness, pale face, tiredness, sudden cold sweat, cold limbs, weak breathing, pale and green complexion, blue lips, low blood pressure, or obnubilation. If these symptoms occur, the needles should be removed as soon as possible. The patient is then kepthorizontal with feet slightly raised, and offered warm sugar water. If the symptoms are serious, DU-26 (Shui Gou), DU-25 (Su Liao), PC-6 (Nei Guan), and ST-36 (Zu San Li) points are selected as emergency points. If the symptoms persist, other professional hospital emergency measures may need to taken.

2. Convulsions

All acupuncturists must determine whether the participants have a history of convulsion.If a patient has a convulsion history during acupuncture treatment, close observation is needed. The practitioner will remove all the needles and take some emergency measures. If the disease is not immediately controlled or the convulsions persist, the participants must be transferred to the emergency center immediately.

3. Severe pains

Severe pains during and after treatment are usually caused by improper needle manipulation or strong stimulation. If the pain is of low intensity, a local compress is used to stop the pain; if the pain is heavier, moxibustion can be applied.

4. Needle sticking

If needle sticking occurs, the patients will be required to relax. If the stickingis caused by twisting the needle inone direction, the problem is solved by rotating the needle in the opposite direction. If the sticking is caused by the over-contraction of the muscle, the needles are removed and the local area of the acupuncture points is pressed, where the needle is stuck, or another needle is placed in the local area, to distract the patients’ attention. If the situation occurs because of a change in the patients’ position, it will be relieved when the patient resumes the correct position.

5. Local infections

If a strict aseptic operation is ignored, local infection occurs. Once the infection is diagnosed, proper treatment will be needed or drug therapy is suggested.

6. Subcutaneous hematoma

During the acupuncture treatment, swelling and bruising may occur when subcutaneous tiny vessels are broken. Ice and local pressure are used to stop the bleeding; at a later stage, a hot compress can be applied and herbs used, to promote blood circulation and remove the blood stasis.

#### Assessment of adverse events or reactions

If other adverse events or reactions occur during the study process, the investigator should analyze the situation, in particular:

1. The temporal relationship between the acupuncture and the adverse event, and whether there is aconnection.

2. Whether the adverse event or reaction is relieved on stopping the treatment.

3. Whether the adverse event or reaction occurs again, when repeating the acupuncture treatment, on the condition of ensuring the patient’s security and consent.

4. Whether the adverse events or reactions can be explained by the patient’s disease progress, a newly occurring disease, or other therapies self-administered by the patients.

#### Reportingadverse events orreactions

Any adverse events or reaction should be recorded in the appropriate form in the CRF. The adverse events will be reported to the principal investigator in Guangdong Provincial hospital and the result of the assessment will be recorded.

### Data collection and outcome assessment

The data required for evaluating the effectiveness of the treatment will be collected at baseline, at the start of the treatment, 1 month after the treatment has begun, 2 months after the treatment has begun, and at the end of the treatment. The data required for the blind method cognitive evaluation will be collected after the second acupuncture treatment and after the last treatment.

#### Primary outcomes

The PSQI, to ratesleep disorders, and HAMD, to rate depression, were applied as primary outcome measurements for effectiveness evaluation. The construction validity and reliability of the PSQI to measure changes in sleep qualityhave been proven in previous studies and this scale has been validated for use in China. The PSQI comprises 19 self-rated questions and 5 otherquestions, all grouped into seven component scores (subjective sleep quality, sleep latency, sleep duration, sleep efficiency, sleep disturbance, hypnotic medication use, daytime dysfunction), each weighted equally on a four-point scale, from 0 to 3. The seven component scores are then added to provide aPSQI score. A cutoff score of 5 has been recommended, with scores >5 indicating subjective insomnia [18]. Changes in levels of depression were measured on the Hamilton hetero-evaluation scale. There are three types of HAMD scale, with 17, 21, and 24 items. We used the 24-item HAMD scale. This scale is of proven discriminant validity, reliability, and sensitivity to change; moreover, it has been validated for use in China. According to the scoring standard, a normal score is less than 8; a score ≥8 and <20 indicates thatthe patient may have depression; a score of ≥20 and ≤35 indicates thatthe patient definitely has depression; anda scorehigher than 35 indicates that the patient has severe depression. In general, the higher the score, the more severe the depression [19].

#### Secondary outcomes

1. Combined medication record. Drugs such as antidepressants and hypnotics medication consumed during the trial (whether or not prescribed by the patient’s doctor) will be recorded and measured on a special scale. Antidepressants, analgesics, and NSAIDs consumed (whether or not prescribed by the patients doctor) atrandomization and at follow up will be documented. These will be measured on a four-point scale: 0=no medication; 1=less than the usual dose; 2=daily, at the usual dose,3=greater than the usual dose.

2. Blind method cognitive evaluation.

3. Baseline variables of patients such as age, sex, education level, profession, income level, weight (kg) and height (cm).

4. A record will be made of any side effects and possible adverse reactions arising from the treatment.

### Data storage and statistical analysis of the trial outcomes

The data compilation form containing the variables of interest will be completed by the corresponding researcher at each center. Data will be documented in the paper CRF by researchers or evaluators and will be double-checked by quality inspectors. The supervisor will inspect the progress of the trial at regular intervals in each branch center. All the detailed original data (paper CRF) will be recorded timely, clearly, and completely. The case reports will be modified only by researchers who are documenting the data. The correct method of modification is tomark a line through the original record and write the revised data next to the original data,so that the change may be clearly visible. The modified data will be documented with data and the initials of the investigator. All the materials will be kept for 5 years after the completion of the study. At each center, the information obtained will be recorded on an electric database by EpiData software 3.1 at the same time, for subsequent statistical analysis.

Predictive Analytics Software (PASW, version18.0) is used for data analyses. Exploratory analysis will include the minimum, maximum, median, mean, and standard deviation. For measurement data, a normality test is necessary. Furthermore, baseline categorical data, demographic characteristics, such as sex and age category, and numerical data are analyzed with chi-squaretests and one-way analyses of variance (ANOVA). Student’s *t*tests are used tocompare different periods of measurement data. Wilcoxon matched-pairs signed-rank tests are used for ranked data. Independent Student’s *t*tests and 95% confidence intervals are used to detect differences between groups of the measurement data only if the normality test and analysis of variancetestsprove significant, otherwise rank-sum tests should be used, which are also used to detect differences between groups of ranked data. Linear mixed models (for continuous outcome variables) and generalized estimating equations (for categorical outcome variables) are used to examine changes over time and the severity of PSQI and HAMD. Both intention-to-treat and per-protocol analyses are used. Data from participants who do not violate the treatment protocols will be included in the per-protocol analysis. Missing data are dealt with using the ‘last value carried forward’ method.

### Study organization

Statistical analysis will be carried out by the Design,Measurement and Evaluation in Clinical Research (DME) professionalsin Guangzhou University of TCM, after approval of the protocol by the Ethics Committee of Guangzhou Provincial Hospital of TCM, 2008GL-26, 2009-07-01. The trial is internationally registered at Chinese Clinical Trial Register http://www.chictr.org/ (ChiCTR-TRC-00000481). A training working team will be established, consisting of DME professionals of Guangzhou University of TCM, staff of the scientific research department and staff of the General Acupuncture Department. The principle institution is Guangdong Provincial Hospital of TCM and the cooperative institutions are the Second People’s Hospital of Zhao Qing city and the People’s Hospital of Hui Zhou. Data management and statistical analysis will be carried out under the guidance of DME professionals according to a pre-specified statistical analysis plan. The clinical trial quality control group will be established, and the investigator in each branch center will be responsible for quality control.

## Discussion

Depression has various clinical manifestations and insomnia is one of the most frequently occurring symptoms. Clinically, we are not simply using regulating spirit and regulating liver acupuncture formula. The formula has been optimized. Lie Que (Lu-7) and Zhao Hai(Kid-6) are two of the confluent points. The actions of these two points can be best understood in relation to the regulation of various meridians, three Jiao, and relative organs. These two empirical points are used as the main points and embedded needles are buried to consolidate the curative effects of the acupuncture.

## Trial status

The project was concluded in the 2011 and we expect to publish the result at the end of 2013.

## Abbreviations

ANOVA: Analysis of variance; CRF: Case report file; DME: Design, measurement and evaluation in clinical research; HAMD: Hamilton rating scale for depression; NSAID: Nonsteroidal anti-inflammatory drug; PASW: Predictive analysis software; PSQI: Pittsburgh sleep quality index; SRICTA: Standards for reporting interventions in clinical trials of acupuncture; TCM: Traditional Chinese medicine.

## Competing interests

The authors declare that they have no competing interests.

## Authors’ contributions

YFC and JHL are responsible for the study design, definition of the primary outcomes, secondary outcomes, sample size calculation, and preparation of the protocol. NGX, SJX, ZHX, and WBF are responsible for the protocol alternation and consummation. All the authors have critically reviewed and approved the final version of the manuscript. WBF had final responsibility for the decision to submit for publication.

## References

[B1] Xu JM Somatization and somatoform disorders Zhongguo Xingwei Yixue Kexue 2004 13 3 359

[B2] van Wingen GA van Eijndhoven P Tendolkar I Buitelaar J Verkes RJ Fernández G Neural basis of emotion recognition deficits in first-episode major depression Psychol Med 2010 8 1 9 10.1017/S0033291710002084 21054920

[B3] Fava M Daytime sleepiness and insomnia as correlates of depression J Clin Psychiatry 2004 65 Suppl 16 S27 S32 15575802

[B4] Wichniak A Wierzbicka A The effects of antidepressants on sleep in depressed patients with particular reference to trazodone in comparison to agomelatine, amitriptyline, doxepin, mianserine and mirtazapine Pol Merkur Lekarski 2011 31 181 65 70 21870714

[B5] Sunderajan P Gaynes BN Wisniewski SR Miyahara S Fava M Akingbala F DeVeaugh-Geiss J Rush AJ Trivedi MH Insomnia in patients with depression: a STAR*D report CNS Spectr 2010 15 394 404 20625372 10.1017/s1092852900029266

[B6] Bolge SC Joish VN Balkrishnan R Kannan H Drake CL Burden of chronic sleep maintenance insomnia characterized by nighttime awakenings among anxiety and depression suffers: results of a national survey Prim Care companion J Clin Psychiatry 2010 12 2 PCC.09m00824 20694118 10.4088/PCC.09m00824gry PMC2911002

[B7] Pigeon WR Hegel M Unützer J Fan MY Sateia MJ Lyness JM Phillips C Perlis ML Is insomnia a perpetuating factor for late-life depression in the IMPACT cohort? Sleep 2008 31 4 481 488 18457235 10.1093/sleep/31.4.481 PMC2279755

[B8] Kaptchuk TJ Acupuncture: theory, efficacy, and practice Ann Intern Med 2002 136 374 383 11874310 10.7326/0003-4819-136-5-200203050-00010

[B9] Sherman KJ Cherkin DC Eisenberg DM Erro J Hrbek A Deyo RA The practice of acupuncture: who are the providers and what do they do? Ann Fam Med 2005 3 2 151 158 10.1370/afm.248 15798042 PMC1466855

[B10] MacPherson H Sinclair-Lian N Thomas K Patients seeking care from acupuncture practitionersin the UK: a national survey Complement Ther Med 2006 14 1 20 30 10.1016/j.ctim.2005.07.006 16473750

[B11] Wang H Qi H Wang BS Cui YY Zhu L Rong ZX Chen HZ Is acupuncture beneficial in depression: a meta-analysis of 8 randomized controlled trials? J Affect Disord 2008 111 2–3 125 134 18550177 10.1016/j.jad.2008.04.020

[B12] Smith CA Hay PP Acupuncture for depression Cochrane Database Syst Rev 2005 2 CD004046 15846693 10.1002/14651858.CD004046.pub2

[B13] Smith CA Hay PP Macpherson H Acupuncture for depression Cochrane Database Syst Rev 2010 1 CD004046 10.1002/14651858.CD004046.pub3 20091556

[B14] Shi XM Acupuncture and Moxibustion 2002 Beijing: Traditional Chinese Medicine publishing house 166 167

[B15] Chinese Society of Psychiatry Chinese Medical Association. Chinese Classification and Diagnostic Criteria of Mental Diseases 2001 3 Jinan: Shandong Science and Technology Press

[B16] American Psychiatric Association Diagnostic and Statistical Manual of Mental Disorders 1994 4 Washington DC

[B17] MacPherson H Altman DG Hammerschlag R Youping L Taixiang W White A Moher D on behalf of the STRICTA Revision Group Revised Standards for Reporting Interventions in Clinical Trials of Acupuncture (SRICTA): Extending the CONSORT statement PLoS Med 7 6 e1000261 10.1371/journal.pmed.1000261 20543992 PMC2882429

[B18] Liu XC Tang MQ Hu L Reliability and validity research of Pittsburgh quality index Chin J Psychiatry 1996 29 2 103 107

[B19] Zhang MY Scales in psychiatric settings 2nd edition Hunan 1998 Hunan Science and Technology Publishing house 121 126

